# Editorial: The psychological endeavors contributing to approach 95-95-95 HIV/AIDS goals

**DOI:** 10.3389/fpsyg.2024.1345243

**Published:** 2024-03-13

**Authors:** Honghong Wang, Xuelin Xiao, Kaili Zhang, Ketema Bizuwork

**Affiliations:** ^1^Central South University, Changsha, Hunan, China; ^2^Xuzhou Medical University, Xuzhou, Jiangsu, China; ^3^Addis Ababa University, Addis Ababa, Ethiopia

**Keywords:** HIV, AIDS, psychological endeavors, 95-95-95 HIV/AIDS goals, WHO goal in 2030

The Joint United Nations Program on HIV/AIDS (UNAIDS) has proposed the ambitious 95-95-95 target to end the HIV epidemic by 2030. Countries worldwide are working hard to achieve this target, while barriers persist in the path to ending AIDS. To better understand how the barriers impact the three 95s, namely, that 95% of all people living with HIV should know their HIV status, 95% of all people with diagnosed HIV infection should receive sustained antiretroviral therapy, and 95% of all people receiving antiretroviral therapy should have viral suppression, this Research Topic focused on the psychological mechanisms under the potential barriers. In total, six articles were published. The factors covered mental health conditions such as depression, change in declarative memory, gender-based concerns, stigmatized reasons for HIV transmission, and the impact of food insecurity on the receipt of care, retention in care, and viral suppression among people living with HIV/AIDS.

The first article by Chen and Barbour, which used an unusual methodology, looked at gender-based concerns among Asian men and women living with HIV. According to the study, Asians with HIV/AIDS are extremely concerned about the prospect of an unintentional revelation of their HIV-positive test result, which could result in psychological stress or sadness. Men were more likely than women to express this anxiety-filled concern, indicating that men require more social support than women do. According to the study's findings, it should be routine practice for local healthcare practitioners to assess a patient's mental status, particularly how it is viewed.

The second article by Arends et al. adopted a behavioral intervention prospective study design focusing on men who have sex with men (MSM) living with HIV and who persistently engage in sexual transmission risk behavior. The study examined the effects of psychological therapy to enhance impulse control and lessen obsessive sexual behavior. The study discovered a significant prevalence of STIs, including HIV, among MSM, particularly those who had engaged in risky activities like chemsex and drug use. The study comes to the conclusion that educational interventions involving multiple professional collaborative interpolations among those engaging in risky behavior for the transmission of STIs, including HIV, improve patient care, mental health, and self-control and lessen societal public health concerns involving these populations.

The third study by Fitzgerald et al. was wisely carried out by multicenter prospective cohort research of women living with HIV, investigating factors predicting unfavorable change in declarative memory change in women living with HIV. The study discovered racial and HIV serostatus-dependent, clinically significant subgroups of women who experience unique phenotypes of declarative memory impairment.

The fourth article by Du et al. study employed bibliometric analysis of articles published in the Web of Science between 1999 and 2022 to investigate global trends in depression among HIV-positive patients. The study found that the growing neuropsychiatric disorders, particularly depression, alcoholism, and drug misuse, which are major risk factors for suicide among PLWH, are responsible for the rising burden of disease worldwide. Additionally, the rise in these neuropsychiatric conditions leads to a rise in non-adherence to ART medications, which leads to virologic failure and poor treatment outcomes in PLWH. The study therefore recommended a program for alcohol and depression screening and treatment, which would ultimately lessen comorbidities such as anxiety and other substance usage among PLWH. In addition, the study highlighted ART drug adherence, mental health, substance misuse, stigma, men who have sex with men, and South Africa as the main factors linked to trends of neuropsychiatric disorder: depression among PLWH internationally.

The fifth article by Uthis et al. employed a two-stage study design to create a standardized instrument for measuring IHS among Thai people living with HIV/AIDS. With regard to HIV-related stigma, the study presented a new set of standardized metrics for IHS. The updated version of the Thai IHS was also employed as an alternative-because of its strong psychometric properties. The recently created study instrument will be employed to assess Thailand's continuing IHS reduction program and the country's national HIV-related stigma monitoring efforts, as well as those of its neighbors Myanmar, Laos, and Cambodia.

The sixth article by Bleasdale et al. is unique in that it examined the effects of food insecurity on receiving care, staying in care, and viral suppression among people living with HIV/AIDS in the United States using a standard study design. The study discovered that food insecurity is a factor in PLWH for not staying in care, which prevents the suppression of the viruses. Therefore, the study recommended an intervention that targets food insecurity as a means of providing effective HIV care.

## Author contributions

KB: Conceptualization, Validation, Writing – original draft, Writing – review & editing. XX: Conceptualization, Validation, Writing – original draft, Writing – review & editing. HW: Conceptualization, Data curation, Supervision, Validation, Writing – original draft, Writing – review & editing. KZ: Conceptualization, Validation, Writing – original draft, Writing – review & editing.

